# A multidisciplinary performance improvement rotation in an internal medicine training program

**DOI:** 10.5116/ijme.5765.0bda

**Published:** 2016-06-29

**Authors:** Srikrishna V. Malayala, Khalid J. Qazi, Abdul J. Samdani, Mushtaq Bhat

**Affiliations:** 1 University at Buffalo/Catholic Health System Internal Medicine Training Program, Buffalo, New York, USA

**Keywords:** Multidisciplinary, performance improvement rotation, internal medicin, training program

## Introduction

Performance Improvement (PI) in health care is defined as “combined and unceasing efforts of everyone - healthcare professionals, patients and their families, researchers, payers, planners and educators - to make the changes that will lead to better patient outcomes, better system performance and better professional development”.^1^  Emphasis on quality improvement with formal rotations in residency programmes is a recent phenomenon.

Our Internal Medicine Training Program based at Sisters of Charity Hospital is a part of the State University of New York at Buffalo (SUNY Buffalo). We have a total of 37 medical residents each year. In 2006, we implemented a four-week rotation titled ‘Performance Improvement Rotation’ and focused on five specific areas: Core measures, Departmental peer review process, Clinical documentation, Patient safety goals and adverse event reporting. The four-week rotation, mandatory for the residents in second year of training, started with an orientation and concluded with a written examination with required performance at 75 percentile.

While many programmes have their own curricula for patient safety rotations, in June 2012, the Accreditation Council for Graduate Medical Education (ACGME) established Clinical Learning Environment Review (CLER) as a component of the Next Accreditation System (NAS) in an attempt to focus on patient safety education and quality improvement in training programmes.^2^^,^^3^ ACGME is the body responsible for accrediting the majority of graduate medical training programmes for physicians in the United States. When ACGME established these CLER guidelines, we decided to evaluate and modify the existing curriculum.

### What was the curriculum?

In the review of core measures, residents understood the Centers for Medicare & Medicaid Services (CMS) core measures.^4^ They performed chart review of patients admitted to the hospital. Core measures are specific clinical measures that, when viewed together, permit an assessment of the quality of care provided in a specific condition, for example, acute myocardial infarction. As a part of Peer Review Process, residents participated in a peer review module to identify and prevent patient safety deficiencies.^5^ To improve and standardize the documentation, the clinical documentation consultants discussed documentation requirements with chart review on the floors with the residents.^6^ Patient safety goals component of the rotation was diverse and included verbal order chart review, medication reconciliation, Do Not Use (DNU) abbreviations, patient identification guidelines as defined by Joint Commission on Accreditation of Healthcare Organizations (JCAHO)^7^ and look alike and sound alike medications. Adverse event reporting exercise had activities like participating in ‘Root Cause Analysis (RCA)’ of few patients.

### How did we change it?

We sent a survey to residents and alumni of the program, and the survey included questions questioning the overall efficiency of the rotation; about its individual components and whether the rotation was useful in private practice following graduation.

### What did we learn?

Majority of the residents agreed that the rotation enhanced their competency as a physician and felt that the rotation met its objectives.

Core measure review was the most appreciated component of the curriculum. Residents responded that their knowledge about core measures improved after this rotation. It was apparent, however, that majority of second-year residents were well versed with core measures and felt that the exercise would be more beneficial if initiated in the first year.

Departmental peer review process was considered equally beneficial. Clinical Documentation review was also uniformly rated superior, and most of the residents agreed that this was a very useful exercise. They recommended that the exercise should be implemented in the first year to improve the documentation skills from beginning of the training. Adverse significant event reporting was considered educational and scored equally high. Root cause analysis review and application was considered comprehensive, and patient safety review curriculum was considered educational.

 Majority of the residents agreed that the rotation should be partially moved to the first year of training. They suggested inclusion of rotations in pharmacy and infection control departments. Graduates felt that the overall information they learnt in the rotation was useful in their current practice. The rotation was looked favourably by their employers and was of benefit in their job interviews. 

### What did we change?

We performed this survey to  answer a major question-Is this rotation beneficial and educational to the residents? And the answer from our residents was a resounding yes.

Based on the feedback from the graduates, we designed a new curriculum and implemented it with substantial changes in 2013. One of the significant changes was splitting the rotation into two mandatory rotations: 2 weeks in the first year of training (titled Quality 101) and 2 weeks in the second year of training (titled Quality 201). This revised curriculum includes all the previous components and incorporates new disciplines of pharmacy, infection control and case management.

Apart from the previous components of core measure chart review, clinical documentation and patient safety measures, Quality 101 included the rotations in Infection control department, pharmacy department and care management.

Isolation protocols like airborne precautions, hand hygiene, droplet and contact precautions, central line-associated bloodstream infection and catheter-associated urinary tract infections prevention techniques, standards for safe disposal were a part of Infection control curriculum. Rotation with pharmacy department includes concepts like how look alike/sound alike orders are handled by pharmacy and similar systematic methods. Care management rotation with the care managers and social workers was included in the rotation as well.

Quality 201 included the previous curriculum about peer review process and adverse event reporting. Based on the feedback from the survey, core measure chart review and clinical documentation were continued in second year as well. The other components added to Quality 201 were care management rounds on medical and surgical floors, rotation with infection control department for Ventilator Associated Pneumonia (VAP) prevention and Surgical Site Infection (SSI) prevention. At the end of both the two-week rotations, the residents take an exit examination that evaluates their PSQI competency.

## Conclusions

A comprehensive report by the Institute for Healthcare Improvement (IHI) and ACGME titled ‘Involving Residents in Quality Improvement: Contrasting “Top-Down” and “Bottom-Up” Approaches’ identified the different approaches of resident involvement in quality improvement activities and the steps that training programmes should take to integrate the residents in such activities.^2^ The IHI report stated that programmes should use three primary drivers for creating a successful quality improvement curriculum- effective curriculum, role models and mentors, appeal of these projects to the residents and infrastructure that embeds these projects in residents’ day to day activities.

We believe that our rotation addresses all three recommendations effectively. We strongly believe that the new PSQI rotation will equip our residents with the latest knowledge and skills required to succeed in the ever evolving and challenging world of Quality Improvement.

### Conflicts of Interest

The authors declare that they have no conflict of interest.
